# Two new species of glyptosternine catfish of the genus *Sineuchiloglanis* (Teleostei, Siluriformes, Sisoridae) from the upper Yangtze River drainage, China

**DOI:** 10.3897/zookeys.1268.173139

**Published:** 2026-02-04

**Authors:** Hai-Min Lv, De-Kui He, Chun-Yun Lei, Fei Liu

**Affiliations:** 1 Institute of Hydrobiology, Chinese Academy of Sciences, No. 7 Donghu South Road, Wuchang District, Wuhan 43007, China Institute of Hydrobiology, Chinese Academy of Sciences Wuhan China; 2 University of Chinese Academy of Sciences, No. 1 Yanqi Lake East Road, Huairou District, Beijing 101408, China University of Chinese Academy of Sciences Beijing China; 3 Yunnan Institute of Fishery Sciences Research, No. 25 Dianchi Road, Xishan District, Kunming, Yunnan 650111, China Yunnan Institute of Fishery Sciences Research Kunming China

**Keywords:** Baishui River, Chishui River, morphology, phylogenetic analysis, *
Sineuchiloglanis
*

## Abstract

Two new species of the genus *Sineuchiloglanis* are described from two tributaries of the upper Yangtze River, China. These two new species can be distinguished from all congeners by morphological comparisons and phylogenetic analysis. *Sineuchiloglanis chishuiensis***sp. nov**. can be distinguished from its congeners by the combination of the following characters: eye enlarged, with diameter 6.8–9.1% of head length (HL); interorbital width 24.1–24.8% of HL; maxillary barbel length 67.1–84.4% of HL; caudal-peduncle depth 27.1–40.3% of caudal-peduncle length; dorsal-fin rays i, 5^1/2^; anal-fin rays i, 4^1/2^; caudal-fin rays i, 7+8, i; gill opening extending to the base of second to third pectoral-fin elements; tip of nasal barbel not reaching anterior orbital margin; pelvic-fin tip reaching or extending beyond anus; and the abdomen slightly convex in profile. *Sineuchiloglanis baishuiensis***sp. nov**. can be distinguished from its congeners by the combination of the following characters: head depth 42–48.4% of HL; maxillary barbel length 65.9–82.1% of HL; caudal-peduncle depth 25.9–40.6% of caudal-peduncle length; dorsal-fin rays i, 5^1/2^; anal-fin rays i, 4^1/2^; caudal-fin rays i, 7+8, i; gill opening extending to the base of the fourth to fifth pectoral-fin elements; tip of maxillary barbel not reaching the lower corner of the gill opening; tip of nasal barbel not reaching anterior orbital margin; and the abdomen flattened in profile. Molecular phylogenetic analysis inferred from mitochondrial *Cytb* gene sequences supported the validity of these two new species.

## Introduction

The genus *Sineuchiloglanis* Li et al., 2022, a member of the subfamily Glyptosterninae within the family Sisoridae of Siluriformes, was established with *Pareuchiloglanis chui* (Li et al., 2020) as the type species from the upper Yangtze River, China by Li et al. (2022). *Sineuchiloglanis* is distinguished from the closely similar genera of Glyptosterninae by the following combination of characteristics: the premaxillary tooth band with lateral sides not extended posteriorly (vs both sides of premaxillary teeth band extend posteriorly in *Chimarrichthys* Sauvage, 1874), the premaxillary tooth patches confluent in one piece with a small median incision in the anterior edge (vs the premaxillary tooth patches contacting, not confluent, divided into two parts in *Creteuchiloglanis*Zhou et al. 2011); the absence a sulcus between the lower lip and the barbel, posterior end of adipose-fin base some distance away from the caudal-fin base, the outer margin of the caudal fin truncated or slightly concave (vs with a sulcus, adipose-fin base fused with caudal-fin base, the outer edge of the caudal fin convex in *Pareuchiloglanis* Pellegrin, 1936 and *Barbeuchiloglanis* Li et al., 2022); and a small gill opening extending only to the second pectoral-fin element (vs extending to the base of the first pectoral-fin element in *Tremeuchiloglanis* Li et al., 2022). Other characteristics of this genus include an interrupted and homodont post-labial groove, and conical and pointed oral teeth (Li et al. 2020, 2022).

All present members of *Sineuchiloglanis* were once part of the genus *Pareuchiloglanis*, and were widely distributed in the upper Yangtze River, Lancang River (the upper Mekong River), and the Ma River (Song Ma in Laos and Vietnam) basins (Li et al. 2022). To date, nine species are valid: five from the upper Yangtze River, viz. *S anteanalis* Fang et al, 1984, *S. chui* Li et al., 2020, *S. hupingshanensis* Kang et al., 2016, *S. robusta* Ding et al., 1991 and *S. sichuanensis* Ding et al., 1991, three from the Lancang River viz. *S. gracilicauda* Wu & Chen, 1979, *S. myzostoma* Norman, 1923 and *S. prolixdorsalis* Li et al., 2007 and one, *S. nebulifera* Ng & Kottelat, 2000 from the Ma River.

Glyptosterniod catfish are typically small to medium-sized benthic fishes adapted to fast-flowing rivers and mountain torrents across southern Asia, including southwestern China, the Indo-China Peninsula, Indo-Burma, the Himalayas, and Central Asia. To thrive in riparian environments, this taxon, alongside other sisorid fishes, has evolved a suite of rheophilic adaptations, including suctorial mouthparts, expanded paired fins, and an adhesive organ (Hora and Silas 1952; Das and Nag 2005). These morphological specializations enable them to adhere to rocky substrates, forage for aquatic invertebrates, and withstand strong currents. Due to such ecological specialization, many species within these taxes exhibit highly restricted distributions (Ng 2006).

Individuals of the two *Sineuchiloglanis* species were collected from the Chishui River, Baishui River, as well as their tributaries in the upper Yangtze River drainage. Through comprehensive morphological comparisons and molecular phylogenetic analysis, these specimens were confirmed as two new species, here described as *S. chishuiensis* sp. nov. and *S. baishuiensis* sp. nov. The key to these two species and five other congeners (*Sineuchiloglanis anteanalis*, *S. hupingshanensis*, *S. robusta*, *S. chui*, *S. sichuanensis*) from the Yangtze River basin is provided.

## Materials and methods

### Sampling

Sample collection was conducted with the authorization of fishery administrative department. Animal experiments and treatments were performed according to the Guide for Animal Care and Use Committee of Institute of Hydrobiology, Chinese Academy of Sciences (IHB, CAS, Protocol No. 2016-018). All specimens were collected as part of routine surveys and killed humanely using approved procedures and preserved in 95% alcohol.

**Figure 1.**
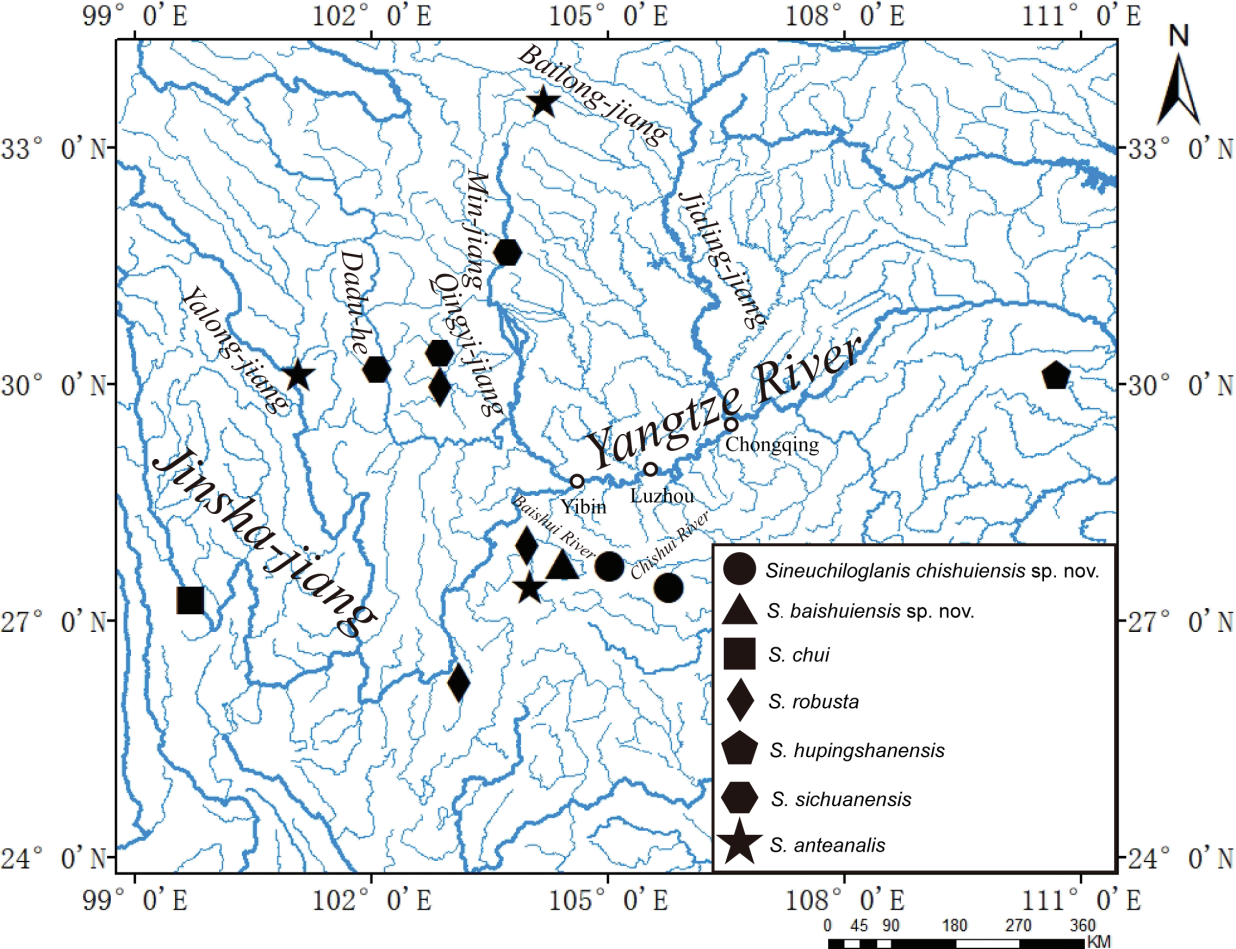
The collection sites of Sineuchiloglanis chishuiensis sp. nov. and Sineuchiloglanis baishuiensis sp. nov.

A total of 48 fish specimens were collected by dipnet from two tributaries of the upper Yangtze River: the Chishui River (24 individuals) and the Baishui River (24 individuals) in Yunnan and Guizhou Province (Fig. 1). The Baishui River, a secondary tributary of the Jinsha River and a primary tributary of the Hengjiang River, is situated in the transitional zone between the Wumeng Mountains and the Sichuan Basin. The Chishui River is a first-order tributary on the right bank of the upper Yangtze River. It remains the only undammed large river in the upper Yangtze basin, retaining its natural flow regime (Xia 2024).

### Morphological methods

Morphological studies were conducted based on both published literature and examination of specimens. All morphological measurements were taken using a dial caliper to the nearest 0.1 mm, from the left side of the specimens whenever possible. All measurements, counts and terminology methods followed Kang et al. (2016). The last branched dorsal-fin ray branches from the base of the dorsal-fin. Subunits of the head are percentages of head length (HL). Head length and measurements of other body parts are given as proportions of standard length (SL). The available samples of *Sineuchiloglanis chishuiensis* sp. nov. and *S. baishuiensis* sp. nov. were compared with specimens of five other *Sineuchiloglanis* species from the upper Yangtze River, i.e., *S. hupingshanensis* (5 specimens), *S. chui* (6 specimens), *S. anteanalis* (25 specimens). Vertebrae counts were conducted using images of microcomputed tomography (Siemens Somatom Definition X-ray machine) reconstructed in CTvox software. The number of vertebrae was expressed as thoracic vertebra (*n*; centrum connecting with rib) plus caudal vertebra (*n*) (Dao et al. 2019).

### Molecular methods

Genomic DNA was extracted from ethanol-preserved pelvic-fin tissues using the salt-extraction method (Aljanabi and Martinez 1997). Mitochondrial cytochrome *b* (*Cytb*) gene sequences were amplified by polymerase chain reaction (PCR) with the primers L14724 (5’-GACTTGAAAAACCACCGTTG-3’) and H15915 (5’-CTCCGATCTCCGGATTACAAGAC-3’) (Xiao et al. 2001). Amplification was performed in a 20 µL reaction volume, containing 10 µL 2*TSINGKE Mix blue, 1 µL DNA template, 1 µL each of forward and reverse primers. Sterile water was added to reach the final volume. The PCR procedures used were as follows: an initial denaturation step at 94 °C for 3 min; a denaturation step at 94 °C for 30 s; annealing at 56 °C for 30 s; extension at 72 °C for 1 min; 35 cycles of the above steps; and a final extension at 72 °C for 10 min. The amplified products were sent to Sangon Biotech (Shanghai) Co., Ltd for purifying and sequenced in both directions with the same primers mentioned above.

Phylogenetic analysis was performed based on 44 newly generated *Cytb* sequences of the two new species and previously published data from NCBI GenBank for an additional 53 sequences of seven species of *Sineuchiloglanis*, six species of *Tremeuchiloglanis*, four species of *Chimarrichthys*, one species of *Bagarius* Bleeker, 1853, five species of *Creteuchiloglanis*, one species of *Exostoma* Blyth, 1980, one species of *Gagata* Bleeker, 1858. One species of *Glaridoglanis* Norman, 1925, one species of *Glyptosternon* M’Clelland, 1842, two species of *Glyptothorax* Blyth, 1980, one species of *Gogangra* Roberts, 2001, three species of *Oreoglanis* Smith, 1933, two species of *Parachiloglanis* Wu, 1981, one species of *Pseudecheneis* Blyth, 1860 and three species of *Pseudexostoma* Chu, 1979. *Akysis brachybarbatus* He & Chen, 1981, *Liobagrus anguillicauda* Nichols, 1926 and *Xiurenbagrus xiurenensis* Zheng, 1981 were included as the outgroup (Table 1). The sequences were manually aligned and revised with MEGA 7 (Kumar et al. 2016). ModelFinder (Kalyaanamoorthy et al. 2017) was used to select the best-fit model using AICc criterion in PhyloSuite. (Zhang et al. 2020). The best-fit model according to AICc was GTR+F+R3. Maximum likelihood phylogenies were inferred using IQ-TREE (Nguyen et al. 2015) under the GTR+R3+F model for 1000 standard bootstraps in PhyloSuite (Zhang et al. 2020). Pairwise genetic distances were calculated using the Kimura-2-parameter (K2P) model (Kimura 1980) in MEGA 7.

**Table 1.** Information on *Sineuchiloglanis* glyptosternine catfishes included in the molecular phylogenetic analysis.

Species	Collection locality	GenBank Accession No.	Data source
*Liobagrus anguillicauda*	Wuyishan, Fujian	AF416888	Peng et al. 2004
*Xiurenbagrus xiurenensis*	Xiuren, Guangxi	DQ192464	Peng et al. 2006
*Akysis brachybarbatus*	Jinghong, Yunnan	AF499603	Peng et al. 2004
*Bagarius yarrelli*	Bizhai, Longlin, Yunnan	HQ322524	Jiang et al. 2011
*Chimarrichthys davidi*	Dujiangyan, Sichuan	AF416883	Peng et al. 2004
*Chimarrichthys kishinouyei*	Tianquan, Sichuan	AY207478	Peng et al. 2004
*Chimarrichthys kishinouyei*	Wenchuan, Sichuan	AY207479	Peng et al. 2004
*Chimarrichthys kishinouyei*	Yinxiu, Dujiangyan, Sichuan	OM428172	Li et al. 2022
*Chimarrichthys longibarbatus*	Yajiang, Sichuan	OM428191	Li et al. 2022
*Chimarrichthys longus*	Amo, Yunnan	DQ192485	Peng et al. 2006
*Creteuchiloglanis gongshanensis*	Yunnan	JN986970	Yu and He. 2012
*Creteuchiloglanis kamengensis*	Chayu, Tibet	AY601767	Guo et al. 2005
*Creteuchiloglanis kamengensis*	Chayu, Tibet	DQ192475	Peng et al. 2006
*Creteuchiloglanis kamengensis*	Chayu, Tibet	AY601768	Guo et al. 2005
*Creteuchiloglanis longipectoralis*	Guolang, Yunlong, Yunan	OM428174	Li et al. 2022
*Creteuchiloglanis longipectoralis*	Guolang, Yunlong, Yunan	OM428175	Li et al. 2022
*Creteuchiloglanis macropterus*	Xiyuegu, Nujiang	KY232889	Kang et al. 2017
*Creteuchiloglanis* sp.	Wayao, Baoshan, Yunnan	OM428176	Li et al. 2022
*Exostoma labiatum*	Beibeng, Tibet	DQ192461	Peng et al. 2006
*Gagata cenia*	Manhaiqiao, Yunnan	DQ192468	Peng et al. 2006
*Glaridoglanis andersonii*	Chayu, Tibet	AY601769	Guo et al. 2005
*Glyptosternon maculatum*	Lhasa, Tibet	AF416891	Peng et al. 2004
*Glyptothorax deqinensis*	Deqin, Yunnan	HQ593590	Jiang et al. 2011
*Glyptothorax sinensis*	Panzhihua, Sichuan	HQ593594	Jiang et al. 2011
*Gogangra viridescens*	—	EU490917	Unpublished
*Oreoglanis delacouri*	—	JN986966	Yu and He 2012
*Oreoglanis macropterus*	Gulang, Yunnan	DQ192479	Peng et al. 2006
*Oreoglanis* sp.	GengmaYunnan	OM428171	Li et al. 2022
*Parachiloglanis drukyulensis*	Chaplaychhu stream, Sarpang Dzongkhag, Bhutan	MG001356	Thoni and Gurung 2018
*Parachiloglanis hodgarti*	Bhutan	MG001355	Thoni and Gurung 2018
*Sineuchiloglanis anteanalis*	Daduhe, Sichuan	KP872692	Ma et al. 2015
*Sineuchiloglanis anteanalis*	—	NC028513	Ma et al. 2015
*Tremeuchiloglanis arcuatum*	Wenliu, Qiubei, Yunnan	OM428177	Li et al. 2022
*Tremeuchiloglanis arcuatum*	Wenliu, Qiubei, Yunnan	OM428178	Li et al. 2022
*Sineuchiloglanis chui*	Judian, Yulong, Yunnan	OM428188	Li et al. 2022
*Sineuchiloglanis gracilicauda*	Kanpu, Weixi, Yunnan	OM428173	Li et al. 2022
*Sineuchiloglanis hupingshanensis*	Hunan Hupingshan National Nature Reserve (HHNNR)	KU356571	Kang et al. 2016
*Sineuchiloglanis chishuiensis* sp. nov.	Zhenxiong, Yunnan	PV974799	This study
*Sineuchiloglanis chishuiensis* sp. nov.	Zhenxiong, Yunnan	PV974800	This study
*Sineuchiloglanis chishuiensis* sp. nov.	Zhenxiong, Yunnan	PV974801	This study
*Sineuchiloglanis baishuiensis* sp. nov.	Zhenxiong, Yunnan	PV974802	This study
*Sineuchiloglanis baishuiensis* sp. nov.	Zhenxiong, Yunnan	PV974803	This study
*Tremeuchiloglanis macrotrema*	Hade, Sanmeng, Lvchun, Yunnan	OM428185	Li et al. 2022
*Tremeuchiloglanis macrotrema*	Tujie, Nanhua, Yunnan	OM428186	Li et al. 2022
*Tremeuchiloglanis macrotrema*	Tujie, Nanhua, Yunnan	OM428187	Li et al. 2022
*Tremeuchiloglanis posteranalis*	Diyu, Guannan, Yunnan	OM428179	Li et al. 2022
*Tremeuchiloglanis posteranalis*	Zhetai, Guannan, Yunnan	OM428180	Li et al. 2022
*Tremeuchiloglanis posteranalis*	Zhetai, Guannan, Yunnan	OM428181	Li et al. 2022
*Tremeuchiloglanis rhabdurus*	Mengdong, Malipo, Yunnan	OM428184	Li et al. 2022
*Sineuchiloglanis robusta*	Ebian, Sichuan	OM428192	Li et al. 2022
*Tremeuchiloglanis salicesbarba*	Lemin, Pancounty, Yunnan	OM428182	Li et al. 2022
*Tremeuchiloglanis salicesbarba*	Lemin, Pancounty, Yunnan	OM428183	Li et al. 2022
*Sineuchiloglanis sichuanensis*	Yajiang, Sichuan	OM428193	Li et al. 2022
*Tremeuchiloglanis sinensis*	Dongshan, Mile, Yunan	OM428194	Li et al. 2022
*Pseudecheneis sulcata*	Beibeng, Tibet	DQ192469	Peng et al. 2006
*Pseudexostoma brachysoma*	Baihualing, Baoshan Prefecture	KU987362	Yang et al. 2016
*Pseudexostoma longipterus*	Qilangdangzhu, Gongshan County	KU987372	Yang et al. 2016
*Pseudexostoma yunnanensis*	Guyong, Tengchong County	KU987382	Yang et al. 2016

## Results

### Sineuchiloglanis chishuiensis sp. nov.

Taxon classificationAnimaliaSiluriformesSisoridae

8152A045-A402-5174-9B09-750A6AC4976E

https://zoobank.org/364FD6AD-EA82-4FD5-93EA-4A3AFAAEA110

Figs 2, 3

#### Type material.

***Holotype*** • IHB 202507021, 113.00 mm SL; Chishui River in the upper Yangtze River drainage in Zhenxiong County, Yunnan Province, China (27°45N, 105°13E; 779 m elevation); August 2024, Liu. F.

**Figure 2.**
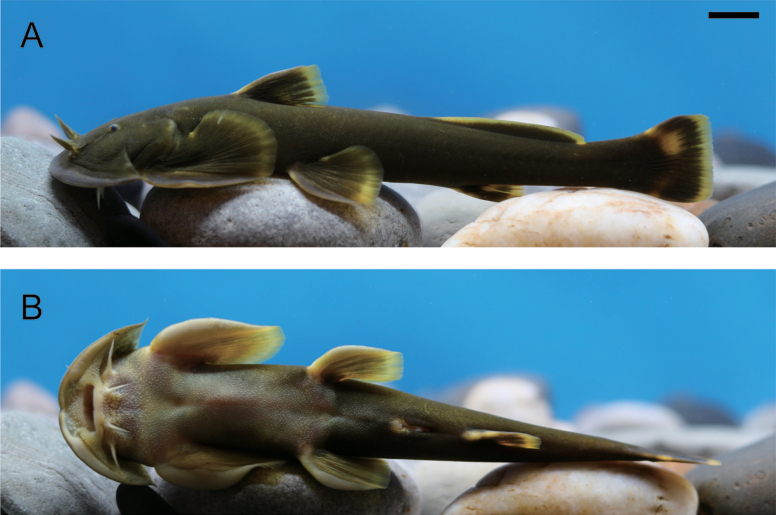
Lateral (**A**) and ventral views (**B**) of live specimen of *Sineuchiloglanis chishuiensis* sp. nov. (IHB202507045). Scale bar: 1 cm

**Figure 3.**
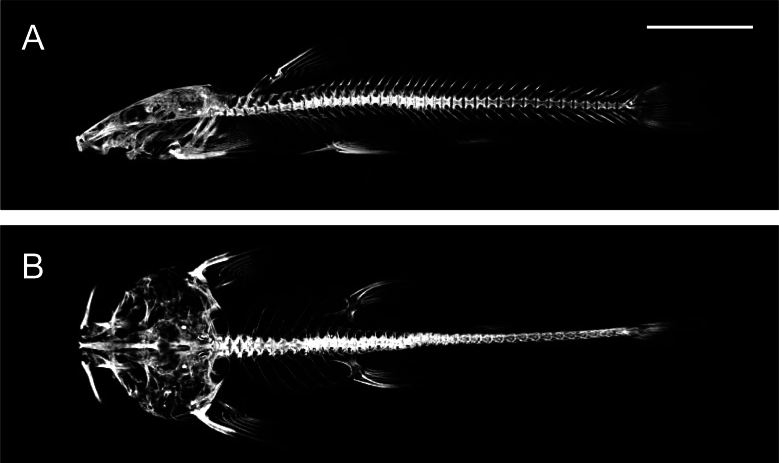
Lateral (**A**) and ventral views (**B**) of the micro-CT graph of the skeletal system of *Sineuchiloglanis chishuiensis* sp. nov. (IHB202507046). Scale bar: 1 cm

***Paratypes*** • IHB 202507003, 1, 84.99 mm SL; Erdao River (a tributary of the Chishui River in the upper Yangtze River drainage) in Bijie City, Guizhou Province, China; March 2023, Liu. F. • IHB 202507001, 202507002, 2, 106.46–121.63 mm SL; Chishui River in the upper Yangtze River drainage in Zhenxiong County, Yunnan Province, China; June 2023, Liu. F. • IHB 202507004–202507011, 202507014, 202507015, 10, 78.98–147.68 mm SL; Tongche River (a tributary of the Chishui River in the upper Yangtze River drainage) in Zhenxiong County, Yunnan Province, China; April 2024, Liu. F. • IHB 202507012, 202507013, 2, 150.15–164.57 mm SL; Chishui River in the upper Yangtze River drainage in Zhenxiong County, Yunnan Province, China; April 2024, Liu. F. • IHB 202507016–202507018, 3, 87.77–134.79 mm SL; Tongche River (a tributary of the Chishui River in the upper Yangtze River drainage) in Zhenxiong County, Yunnan Province, China; September 2024, Liu. F. • IHB 202507019, 202507020, 202507022, 3, 81.12–182.92 mm SL; Chishui River in the upper Yangtze River drainage in Zhenxiong County, Yunnan Province, China; August 2024, Liu. F. • IHB 202507045, 1, 120 mm SL; Chishui River in the upper Yangtze River drainage in Zhenxiong County, Yunnan Province, China; March 2025, Liu. F. • IHB 202507046, 1, 52 mm SL; Tongche River (a tributary of the Chishui River in the upper Yangtze River drainage) in Zhenxiong County, Yunnan Province, China; May 2025, Liu. F.

#### Diagnosis.

**Figure 4.**
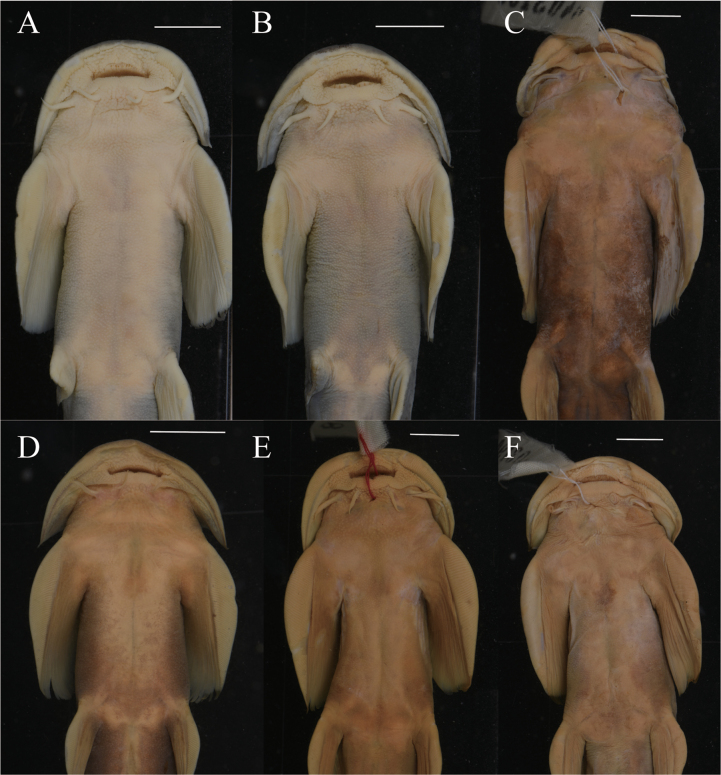
Abdominal features of specimen of *Sineuchiloglanis* from the Yangtze River drainage, China. **A**. *S. baishuiensis* (IHB202507036); **B**. *S. chishuiensis* (IHB202507005); **C**. *S. chui* (0403168); **D**. *S. hupingshanensis* (20080622224); **E**. *S. anteanalis* (0509408); **F**. *S. robusta* (0403190). Scale bars: 1 cm

*Sineuchiloglanis chishuiensis* sp. nov. can be readily distinguished from congeners by the following characters: dorsal-fin rays i, 5^1/2^; pectoral-fin rays i, 12–13; anal-fin rays i, 4^1/2^; caudal-fin rays i, 7+8, i; gill opening extending to the base of second to third pectoral-fin elements; tip of maxillary barbel not reaching or extending beyond the lower corner of the gill opening; tip of nasal barbel not reaching anterior orbital margin; pelvic-fin tip reaching or extending beyond anus; the ventral abdominal surface slightly convex (Fig. 4). And it can be further distinguished from congener by the following characters: eye enlarged, with diameter 6.8–9.1% of HL; head length behind the eyes 35.6–40.3% of HL; interorbital width 24.1–24.8% of HL; maxillary barbel length 67.1–84.4% of HL; caudal-peduncle length 21.5–29.2% of SL; pelvic-fin origin to anal-fin origin length 23.4–28.5% of SL; dorsal-fin insertion to adipose-fin origin length 14.1–23.2% of SL; post-adipose length 8.2–12.7% of SL; caudal-peduncle depth 27.1–40.3% of caudal-peduncle length; pectoral-fin depth 53.0–70.5% of pectoral length. The primary diagnostic characters for species of *Sineuchiloglanis* are summarized in Table 2.

**Table 2.** Major diagnostic characters of *Sineuchiloglanis* species from the Yangtze River, China.

Characters	*Sineuchiloglanis chishuiensis* sp. nov.	*Sineuchiloglanis baishuiensis* sp. nov.	*Sineuchiloglanis hupingshanensis*	*Sineuchiloglanis chui*	*Sineuchiloglanis anteanalis*	*Sineuchiloglanis robusta*	*Sineuchiloglanis sichuanensis*
**Eye diameter (% HL)**	6.8–9.1	6.1–9.2	6.2–7.1	5.2–6.7	6.0–7.9	5.1–7.9	4.1–6.9
**Interorbital distance (% HL)**	24.1–28.8	22.8–29.6	20.4–23.8	23.4–26.0	22.4–25.9	25.4–26.8	27.1–30.0
**Head length behind the eyes (% HL)**	35.6–40.3	35.4–39.4	35.8–42.7	40.4–45.5	34.7–40.6	——	——
**Maxillary barbel length (% HL)**	67.1–84.8	65.9–82.1	73.5–79.6	73.8–84.0	77.1–90.3	90.5–97.7	91.1–96.2
**Head depth (% HL)**	38.9–53.1	42.0–48.4	37.2–41.1	36.4–47.6	34.8–52.1	——	——
**Head width (% SL)**	18.5–24.5	18.1–21.0	18.0–21.2	19.9–22.8	19.6–21.8	21.3–23.9	20.6–23.5
**Caudal-peduncle length (% SL)**	21.5–29.2	20.6–26.7	21.2–26.1	18.7–24.3	22.3–29.2	21.0–25.0	15.7–19.6
**Dorsal-fin insertion to adipose-fin origin distance (% SL)**	14.1–23.2	15.5–25.5	12.1–25.1	15.3–25.7	12.8–20.1	26.5–33.5	25.2–28.4
**Pelvic-fin origin to anal-fin origin distance (% SL)**	24.4–32.4	27.8–33.1	27.2–31.6	29.2–32.5	24.1–28.6	——	——
**Pelvic insertion to adipose origin distance (% SL)**	15.4–19.5	17.3–22.3	13.8–21.3	16.3–21.3	10.5–17.0	——	——
**Post-adipose length (% SL)**	8.2–12.7	8.1–11.2	7.6–10.4	6.7–10.2	7.3–13.7	7.2–9.8	4.6–7.6
**Caudal-peduncle depth (% caudal-peduncle of length)**	27.1–40.3	25.9–40.6	32.7–41.5	21.8–41.3	13.0–24.6	21.9–28.1	37.8–49.2
**Pectoral-fin depth (% pectoral-fin length)**	53.0–70.5	61.0–78.2	38.4–51.4	42.2–56.3	51.0–72.7	——	——
**Pelvic-fin width (% pelvic-fin length)**	43.0–68.4	56.6–76.6	35.6–49.0	42.0–60.0	42.7–74.4	——	——
**Dorsal-fin ray**	i, 5^1/2^	i, 5^1/2^	i, 4^1/2^	i, 4^1/2^	i, 5^1/2^	i, 5^1/2^	i, 5^1/2^
**Pectoral-fin ray**	i, 12–13	i, 12–14	i, 13–14	i, 13–14	i, 13–14	i, 14–15	i, 13–14
**Anal-fin ray**	i, 4^1/2^	i, 4^1/2^	i, 3^1/2^	i, 3^1/2^	i, 3^1/2^	i, 4^1/2^	i, 4^1/2^
**Caudal-fin ray**	i, 7+8, i	i, 7+8, i	i, 7 + 8, i	i, 6 + 7, i	i, 6 + 7, i	i, 7 + 8, i	i, 7 + 8, i
**Vertebra number**	13 + 25 = 38 (*n* = 1); 13 + 24 = 37 (*n* = 1); 12 + 24 = 36 (*n* = 2)	13 + 23 = 36 (*n* = 2); 13 + 24 = 37 (*n* = 2)	13 + 24 = 37 (*n* = 1); 13 + 23 = 36 (*n* = 1); 13 + 25 = 38 (*n* = 2)	12 + 21 = 33 (*n* = 1); 12 + 20 = 32 (*n* = 1); 11 + 21 = 32 (*n* = 2)	12 + 22 = 34 (*n* = 2); 13 + 21 = 34 (*n* = 1); 12 + 21 = 33 (*n* = 1)	12 + 21 = 33 (*n* = 1)	19 + 15 = 34 (*n* = 2)
**Lower corner of gill opening**	Opposite base of the second and third pectoral-fin elements	Opposite base of the fourth and fifth pectoral-fin elements	Opposite base of the fourth and fifth pectoral-fin elements	Opposite base of the second and third pectoral-fin elements	Opposite base of the second and third pectoral-fin elements	Opposite base of the fourth and fifth pectoral-fin elements	Opposite base of the second and third pectoral-fin elements
**Maxillary barbel tip**	Not reaching or extending beyond lower gill-opening corner	Not reaching lower gill-opening corner	Extending beyond lower gill-opening corner	Not reaching or reaching lower gill-opening corner	Reaching or extending beyond lower gill-opening corner	Not reaching lower gill-opening corner	Reaching lower gill-opening corner
**Nasal barbel tip**	Not reaching anterior orbital	Not reaching anterior orbital	Extending beyond anterior orbital	Extending beyond anterior orbital and not reaching posterior orbital	Not reaching or reaching anterior orbital	Not reaching anterior orbital	Extending beyond posterior orbital
**Pelvic-fin tip**	Reaching or extending beyond anus	Not reaching, reaching, or extending beyond anus	Not reaching anus	Reaching or extending beyond anus	Reaching or extending beyond anus	Not reaching or reaching anus (♀); extending beyond anus (♂)	Not reaching anus (♀); reaching anus (♂)
**Ventral abdominal surface**	convex	flattened	flattened	convex	flattened	flattened	–

#### Description.

Morphometric characters of 22 type specimens are shown in Table 3. Dorsal-fin rays i, 5^1/2^; pectoral-fin rays i, 12–13; pelvic-fin rays i, 5; anal-fin rays i, 4^1/2^; branched caudal-fin rays i,7+8, i; vertebra 13 + 25 = 38 (*n* = 1); 13 + 24 = 37 (*n* = 1); 12 + 24 = 36 (*n* = 2) (Table 4).

**Table 3.** Morphometric data for species of *Sineuchiloglanis chishuiensis* sp. nov. and *S. baishuiensis* sp. nov.

	*Sineuchiloglanis chishuiensis* sp. nov. (*n* = 22)				*Sineuchiloglanis baishuiensis* sp. nov. (*n* = 22)			
	Min	Max	Mean	S.D.	Min	Max	Mean	S.D.
**Standard length (mm)**	70.7	162.1	108.6	25.3	74.5	178.8	113.7	35.1
**Percentage of standard length**								
**Predorsal length**	31.2	37.1	33.8	1.5	29.5	37.4	33.7	1.7
**Prepectoral length**	16.3	21.7	19	1.2	16.4	20.2	18	1
**Prepelvic length**	39.1	45	42.1	1.3	39.3	43.8	41.5	1.1
**Pre-anal length**	64.4	71.9	68.5	1.8	67.7	74.1	70.7	1.7
**Dorsal-fin base length**	8.4	10.3	9.3	0.6	7.9	11.4	9.7	0.8
**Body depth**	11.7	18.2	14.4	1.8	11.5	17.4	14.5	1.5
**Body depth at anus**	11.1	17	13	1.5	11.2	17.1	13.7	1.3
**Head length**	19.9	26.4	22.7	1.4	20	24	22	1
**Head width**	18.5	24.5	21.1	1.6	18.1	21	19.9	0.9
**Head depth**	8.8	12.3	10.7	1	8.7	11.4	10.1	0.7
**Pectoral-fin length**	21.1	28.2	24.5	1.8	21.2	29.5	24.4	1.9
**Pelvic-fin length**	15.2	20.5	17.8	1.3	14.5	20.5	17.8	1.3
**Anal-fin base length**	5.6	8.4	6.7	0.7	6	7.7	6.8	0.4
**Caudal-fin length**	12.6	17.4	14.3	1.3	11.9	16.3	13.7	1.2
**Adipose-fin base length**	27.8	32.5	30.2	1.4	24.5	32.8	28.3	2.3
**Dorsal to adipose distance**	14.1	23.2	19.5	2.6	15.5	25.5	21.4	2.5
**Pelvic-fin origin to anal-fin origin length**	23.4	28.5	26.6	1.6	26.1	32	29	1.7
**Pelvic insertion to adipose origin**	15.4	19.5	17.4	1.2	17.3	22.3	19.2	1.2
**Post-adipose distance**	8.2	12.7	9.8	1.3	8.1	11.2	9.4	0.8
**Caudal-peduncle length**	21.5	29.2	24.9	1.8	20.6	26.7	23.2	1.7
**Caudal-peduncle depth**	6.6	9.5	7.8	0.8	6.3	8.7	7.7	0.7
**Percentage of head length**								
**Snout length**	52.8	57.7	55.2	1.2	53.7	57.3	55.4	1
**Interorbital distance**	24.1	28.8	26.5	1.3	22.8	29.6	26	1.8
**Eye diameter**	6.8	9.1	7.7	0.6	6.1	9.2	8	1
**Head length behind the eyes**	35.6	40.3	37.6	1.2	35.4	39.4	37.6	1
**Head depth**	38.9	53.1	47.3	4	42	48.4	45.7	1.6
**Nasal barbel length**	24.9	35	31.3	2.6	23.3	35.1	29.7	2.8
**Maxillary barbel length**	67.1	84.8	77.5	4.5	65.9	82.1	73.7	3.7
**Inner mandibular barbel length**	15.5	20.6	17.7	1.5	14.4	19.1	16.6	1.4
**Out mandibular barbel length**	23.8	30.2	27.4	1.7	23.1	29.2	25.9	1.7
**Percentage of caudal-peduncle length**								
**Caudal-peduncle depth**	27.1	40.3	31.3	3.4	25.9	40.6	33.6	4.5
**Percentage of pectoral-fin length**								
**Pectoral-fin width**	53	70.5	62.7	4.2	61	78.2	67.9	4.9
**Percentage of pelvic-fin length**								
**Pelvic-fin width**	43	68.4	58.2	6	53.6	76.6	61.9	6.1

**Table 4.** Main characteristics for comparison among species of *Sineuchiloglanis* from the Yangtze River drainage, China.

Characters	*Sineuchiloglanis chishuiensis* sp. nov.	*Sineuchiloglanis baishuiensis* sp. nov.	*Sineuchiloglanis hupingshanensis*	*Sineuchiloglanis chui*	*Sineuchiloglanis anteanalis*	*Sineuchiloglanis robusta*	*Sineuchiloglanis sichuanensis*
**Dorsal-fin ray**	i, 5^1/2^	i, 5^1/2^	i, 4^1/2^	i, 4^1/2^	i, 5^1/2^	i, 5^1/2^	i, 5^1/2^
**Pectoral-fin ray**	i, 12–13	i, 12–14	i, 13–14	i, 13–14	i, 13–14	i, 14–15	i, 13–14
**Pelvic-fin ray**	i, 5	i, 5	i, 5	i, 5	i, 5	i, 5	i, 5
**Anal-fin ray**	i, 4^1/2^	i, 4^1/2^	i, 3^1/2^	i, 3^1/2^	i, 3^1/2^	i, 4^1/2^	i, 4^1/2^
**Caudal-fin ray**	i, 7 + 8, i	i, 7 + 8, i	i, 7 + 8, i	i, 6 + 7, i	i, 6 + 7, i	i, 7 + 8, i	i, 7 + 8, i
**Vertebra number**	13 + 25 = 38 (*n* = 1); 13 + 24 = 37 (*n* = 1); 12 + 24 = 36 (*n* = 2)	13 + 23 = 36 (*n* = 2); 13 + 24 = 37 (*n* = 2)	13 + 24 = 37 (*n* = 1); 13 + 23 = 36 (*n* = 1); 13 + 25 = 38 (*n* = 2)	12 + 21 = 33 (*n* = 1); 12 + 20 = 32 (*n* = 1); 11 + 21 = 32 (*n* = 2)	12 + 22 = 34 (*n* = 2); 13 + 21 = 34 (*n* = 1); 12 + 21 = 33 (*n* = 1)	12 + 21 = 33 (*n* = 1)	19 + 15 = 34 (*n* = 2)
**Lower corner of gill opening**	Opposite base of the second and third pectoral-fin elements	Opposite base of the fourth and fifth pectoral-fin elements	Opposite base of the fourth and fifth pectoral-fin elements	Opposite base of the second and third pectoral-fin elements	Opposite base of the second and third pectoral-fin elements	Opposite base of the fourth and fifth pectoral-fin elements	Opposite base of the second and third pectoral-fin elements
**Maxillary barbel tip**	Not reaching or extending beyond lower gill-opening corner	Not reaching lower gill-opening corner	Extending beyond lower gill-opening corner	Not reaching or reaching lower gill-opening corner	Reaching or extending beyond lower gill-opening corner	Not reaching lower gill-opening corner	Reaching lower gill-opening corner
**Nasal barbel tip**	Not reaching anterior orbital	Not reaching anterior orbital	Extending beyond anterior orbital	Extending beyond anterior orbital and not reaching posterior orbital	Not reaching or reaching anterior orbital	Not reaching anterior orbital	Extending beyond posterior orbital
**Pectoral-fin tip**	Not reaching, reaching, or extending beyond pelvic-fin origin	Not reaching or reaching pelvic-fin origin	Not reaching pelvic-fin origin	Not reaching pelvic-fin origin	Reaching or extending beyond pelvic-fin origin	Not reaching pelvic-fin origin	Reaching pelvic-fin origin
**Pelvic-fin tip**	Reaching or extending beyond anus	Not reaching, reaching, or extending beyond anus	Not reaching anus	Reaching or extending beyond anus	Reaching or extending beyond anus	Not reaching or reaching anus (♀); extending beyond anus (♂)	Not reaching anus (♀); reaching anus (♂)

Body compressed, with a slightly raised back. Depth of body 11.7–18.2% of SL, width of body 12.6–17.5% of SL. Head depressed, rostral margin rounded when viewed dorsally. Depth of head 8.8–12.3% of SL, width of head 18.5–24.5% of SL. Body smooth and scaleless, lateral line complete and midlateral. Oral region and anterior part of abdomen with dense papillae, density gradually decreasing posteriorly. Lateral abdominal regions slightly convex, forming a shallow medially invaginated groove along the ventral midline. Caudal fin truncate, with two unbranched and 7+8 branched fin rays (Fig. 3). Dorsal-fin located in anterior third of body, with one unbranched and six branched fin rays without spine, outer margin straight; tip of dorsal-fin rays extending beyond vertical through pelvic-fin base when depressed. Adipose fin located in anterior three-fifths of body, not confluent with caudal fin. Pectoral-fin enlarged, located in anterior one-fifth of body, with one unbranched and 12 branched fin rays; first ray of each broadened; regular striae on ventral surface; tip of pectoral-fin rays not reaching, reaching, or extending beyond pelvic-fin origin. Pelvic-fin enlarged, located in anterior two-fifths of body, with one unbranched and five branched fin rays; first element on each broadened; regular striae on ventral surface; tip of pelvic-fin rays reaching or extending beyond anus. Anal-fin located in anterior two-thirds of body, with one unbranched and five branched fin rays (last branched fin ray branches from the base of the dorsal-fin). Anus located approximately at midpoint of the distance between the pelvic-fin insertion and the anal-fin origin, close to the anal-fin origin.

Mouth wide (mouth width of 32.1–40.2% of HL), inferior, transverse. Premaxillary tooth band with a deep median indentation, sides not extending posteriorly. Dentary tooth band with two short, wide patches. Oral teeth conical, pointed. Barbels four pairs. Maxillary barbel with flap of skin fringing posterior margin, tip pointed, not reaching or extending beyond lower gill opening corner. Lower lip connected to base of maxillary barbel by skin flap, without sulcus between them. Mandibular barbels two pairs, inner mandibular barbel shorter than outer mandibular barbel. Origin of inner mandibular barbel close to midline. Outer mandibular barbel originating posterolateral of inner mandibular barbel. Post-labial groove interrupted, ending at base of inner mandibular barbel. Nasal barbel with small flap of thin skin fringing posterior margin, not reaching anterior margin of orbital. Eye small, almost round, subcutaneous, located on upper lateral surface of head, closer to dorsal-most extremity of gill opening. Gill opening extending to the base of second or third pectoral-fin elements.

#### Coloration.

In fresh specimens, body green-yellow on dorsal surface. On venter, from snout to the pelvic-fin insertion milky white, after pelvic-fin green-yellow. Around the anus pale yellow. Pectoral-fin and pelvic-fin with pale yellow distal margins, lateral green-yellow and medial pale yellow. Dorsal-fin, adipose-fin, and anal-fin green-yellow with pale yellow distal margins. Caudal fin green-yellow with a small pale yellow patch and pale yellow distal margin. In alcohol-fixed specimens, body grey, ventral surface, and margins of fins milky white.

#### Ecology and habitat.

**Figure 5.**
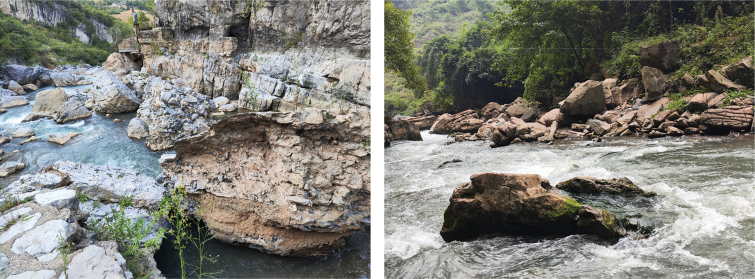
Habitat of *Sineuchiloglanis chishuiensis* sp. nov. in its type locality, Chishui River.

*Sineuchiloglanis chishuiensis* sp. nov. inhabits rapids and riffles, with primarily rocky substrates (Fig. 5). The water temperature was 20.0–26.5 °C during the survey period in August 2024. The species co-occurred with *Schizothorax grahami* Regan, 1904 and *Sinogastromyzon sichangensis* Chang, 1944, in this type locality.

#### Distribution.

This new species is presently only known from the headwaters of Chishui River in the Yangtze River drainage in Zhenxiong County of Yunnan Province, and Bijie City of Guizhou Province, China.

#### Etymology.

The specific epithet is derived from its type locality, Chishui River.

### Sineuchiloglanis baishuiensis sp. nov.

Taxon classificationAnimaliaSiluriformesSisoridae

89040666-B625-5E2D-8592-F6816FE730C0

https://zoobank.org/44FD6E76-F725-4F5F-B5A5-B1AAC137514D

Figs 6, 7

#### Type material.

***Holotype*** • IHB 202507042, 94.47 mm SL; Baishui River in the upper Yangtze River drainage in Zhenxiong County, Yunnan Province, China (27°38N, 104°46E; 791 m elevation); August 2024, Liu F.

**Figure 6.**
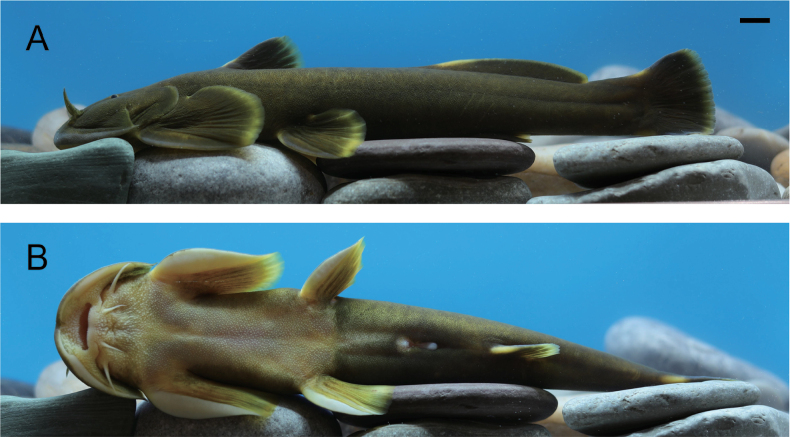
Lateral (**A**) and ventral views (**B**) of a live specimen of *Sineuchiloglanis baishuiensis* sp. nov. (IHB202507047). Scale bar: 1 cm.

**Figure 7.**
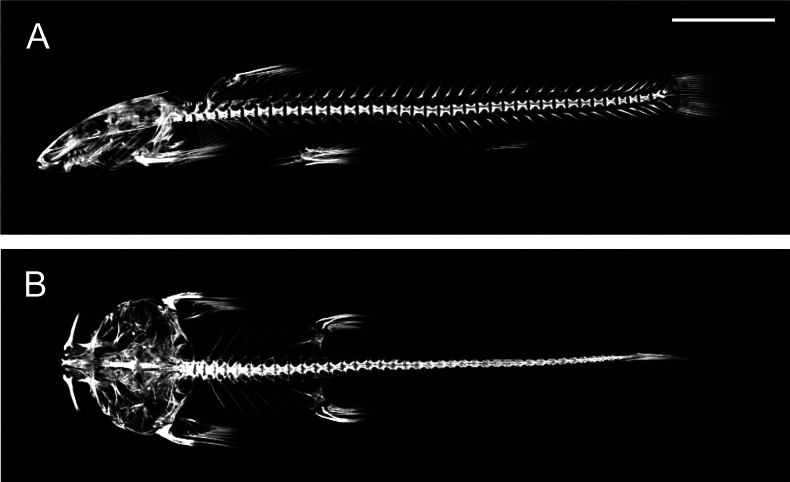
Lateral (**A**) and dorsal (**B**) micro-CT graph of skeletal system of *Sineuchiloglanis baishuiensis* sp. nov. (IHB202507048). Scale bar: 1 cm.

***Paratypes*** • IHB 202507023–202507039, 17, 74.61–178.80 mm SL; Baishui River in the upper Yangtze River drainage in Zhenxiong County, Yunnan Province, China; April 2024, Liu. F. • IHB 202507040, 202507041, 202507043, 202507044, 4, 76.16–146.29 mm SL; Baishui River in the upper Yangtze River drainage in Zhenxiong County, Yunnan Province, China; August 2024, Liu. F. • IHB 202507047, 1, 205 mm SL; Waqiao River (a tributary of the Baishui River in the upper Yangtze River drainage) in Zhenxiong County, Yunnan Province, China; May 2025, Liu. F. • IHB 202507048, 1, 60 mm SL; Waqiao River (a tributary of the Baishui River in the upper Yangtze River drainage) in Zhenxiong County, Yunnan Province, China; August 2022, Liu. F.

#### Diagnosis.

*Sineuchiloglanis baishuiensis* sp. nov. can be readily distinguished from congeners by the following characters: dorsal-fin rays i, 5^1/2^; anal-fin rays i, 4^1/2^; caudal-fin rays i, 7+8, i; gill opening extending to the base of fourth to fifth pectoral-fin elements; tip of maxillary barbel not reaching the lower corner of the gill opening; tip of nasal barbel not reaching anterior orbital margin; the ventral abdominal surface flattened (Fig. 4). And it can be further distinguished from congener by the following characters: head length behind the eyes 35.4–39.4% of HL; head depth 42–48.4% of HL; maxillary barbel length 65.9–82.1% of HL; pelvic insertion to adipose origin 17.3–22.3% of SL; dorsal-fin insertion to adipose-fin origin length 15.5–25.5% of SL; caudal-peduncle length 20.6–26.7% of SL; head width 18.1–21.0% of SL; post-adipose length 8.1–11.2% of SL; pectoral-fin width 61.0–78.2% of pectoral-fin length; pelvic-fin width 53.6–76.6% of pelvic-fin length; caudal-peduncle depth 25.9–40.6% of caudal-peduncle length. The primary diagnostic characters for species of *Sineuchiloglanis* are summarized in Table 2.

#### Description.

Morphometric characters of 22 type specimens are shown in Table 3. Dorsal-fin rays i, 5^1/2^; pectoral-fin rays i, 12–14; pelvic-fin rays i, 5; anal-fin rays i, 4^1/2^; caudal-fin rays i,7+8, i; vertebra 13 + 23 = 36 (*n* = 2); 13 + 24 = 37 (*n* = 2). (Table 4).

Body compressed, with a slightly raised back. Depth of body is 11.5–17.4% of SL, width of body is 14.3–17.3% of SL. Head depressed, rostral margin rounded when viewed dorsally. Depth of head is 8.7–11.4%, width of head is 18.1–21.4% of standard length. Body smooth and scaleless, lateral line complete and midlateral. Oral region and anterior part of abdomen with dense papillae, density gradually decreasing posteriorly. The ventral abdominal surface is flattened. Caudal fin truncate, with two unbranched and 7+8 branched fin rays (Fig. 7). Dorsal-fin located in anterior third of body, with two unbranched and six branched fin rays (the last branched fin ray branches from the base of anal-fin), without spine, outer margin straight; tip of dorsal-fin rays extending beyond vertical through pelvic-fin base when depressed. Adipose fin located in anterior three-fifths of body, not confluent with caudal fin. Pectoral-fin enlarged, located in anterior one-fifth of body, with one unbranched and 13 branched fin rays; first element on each broadened; regular striae on ventral surface; tip of pectoral-fin rays reaching or not reaching pelvic-fin origin. Pelvic-fin enlarged, located in anterior two-fifths of body, with one unbranched and five branched fin rays; first ray of each broadened; regular striae on ventral surface; tip of pelvic-fin rays not reaching, reaching, or extending beyond anus. Anal-fin located in anterior two-thirds of body, with one unbranched and five branched fin rays (last branched fin ray branches from the base of anal fin). Anus is located approximately at midpoint of the distance between the pelvic-fin insertion and the anal-fin origin, close to the pelvic-fin insertion.

Mouth wide (mouth width of 33.0–40.4% of HL), inferior, transverse. Premaxillary tooth band with a deep median indentation, sides not extending posteriorly. Dentary tooth band with two short, wide patches. Oral teeth conical, pointed. Barbels four pairs. Maxillary barbel with flap of skin fringing posterior margin, tip pointed, not reaching the lower corner of gill opening. Lower lip connected to base of maxillary barbel by skin flap, without sulcus between them. Mandibular barbels two pairs, inner mandibular barbel shorter than outer mandibular barbel. Origin of inner mandibular barbel close to midline. Outer mandibular barbel originating posterolateral of inner mandibular barbel. Post-labial groove interrupted, ending at base of inner mandibular barbel. Nasal barbel with small flap of thin skin fringing posterior margin, not reaching anterior margin of orbital. Eye small, almost round, subcutaneous, located on upper lateral surface of head, closer to dorsal-most extremity of gill opening. Gill opening extending to the base of fourth or fifth pectoral-fin elements.

#### Coloration.

In fresh species, body green-yellow on dorsal surface. On venter, from snout to the pelvic-fin insertion milky white, after pelvic-fin green-yellow. Around the anus pale yellow. Pectoral-fin and pelvic-fin with pale yellow distal margins, lateral green-yellow and medial pale yellow. Dorsal fin, adipose fin, and anal fin green-yellow with pale yellow distal margins. Caudal fin green-yellow with a small pale yellow patch and pale yellow distal margin. In alcohol-fixed specimens, body grey, ventral surface, and margins of fins milky white.

#### Ecology and habitat.

**Figure 8.**
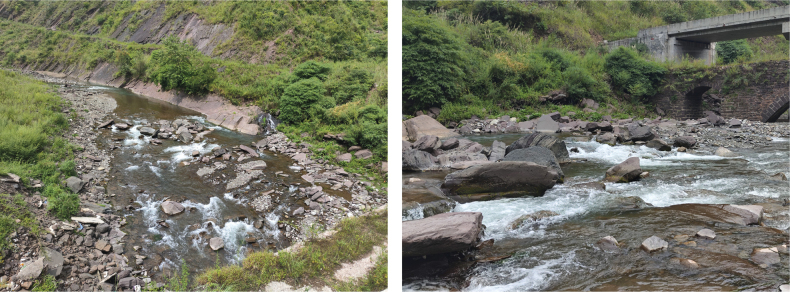
Habitat of *Sineuchiloglanis chishuiensis* sp. nov. in its type locality, Baishui River.

*Sineuchiloglanis baishuiensis* sp. nov. inhabits rapids and riffles, with primarily rocky substrates. (Fig. 8) The water temperature was 19.9 °C during the survey period in August 2024. The species co-occurred with *Schizothorax grahami* in this type locality.

#### Distribution.

This new species is currently known only from the Baishui River in the Yangtze River drainage, in Zhenxiong County, Yunnan Province, China.

#### Etymology.

The specific epithet is derived from its type locality, Baishui River.

##### Molecular phylogenetic analysis

**Figure 9.**
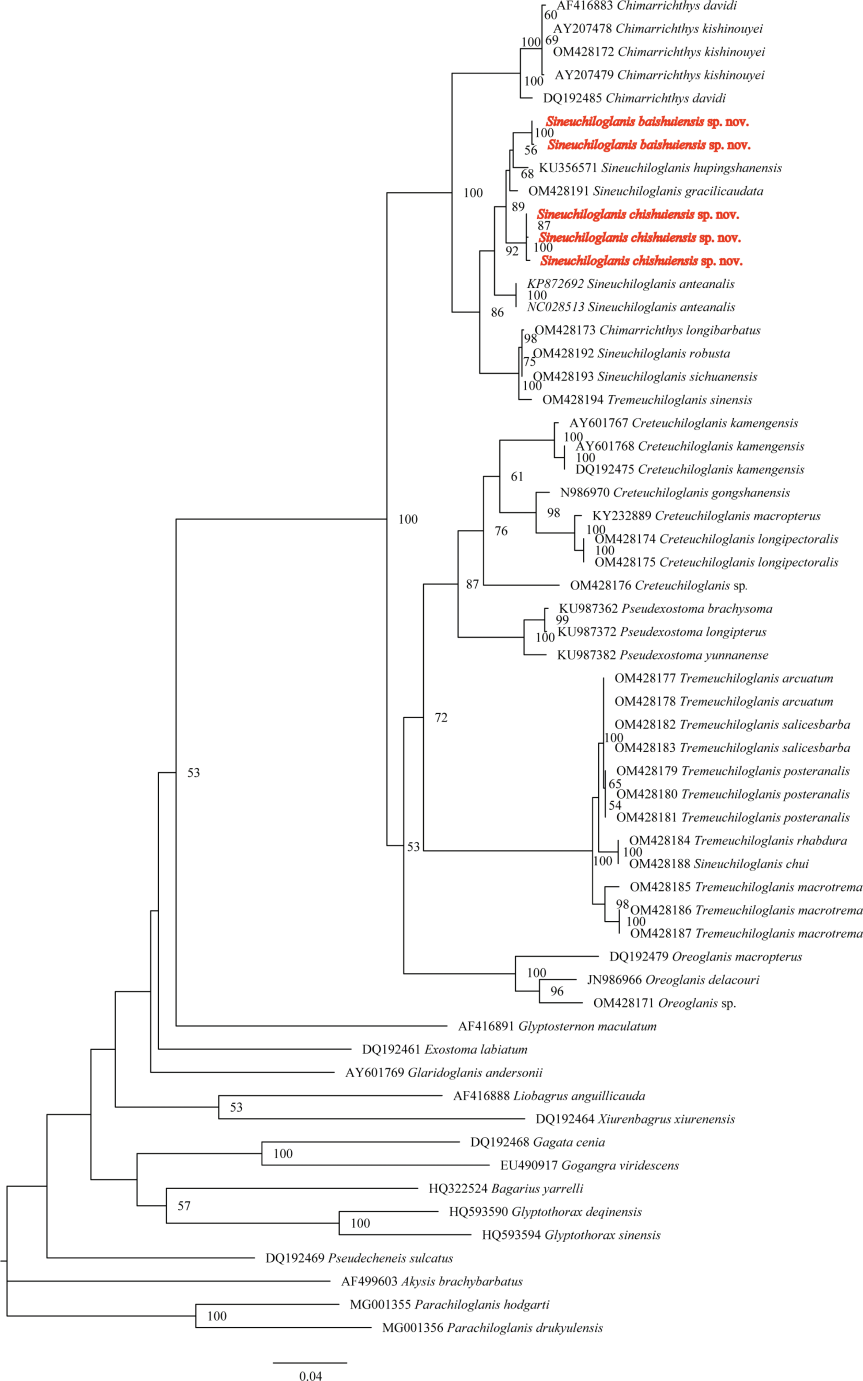
Phylogenetic tree based on maximum likelihood method inferred from mitochondrial *Cytb* gene sequences of *Sineuchiloglanis chishuiensis* sp. nov., *S. baishuiensis* sp. nov. and its closely allied forms. Numbers below the branches represent bootstrap values (> 50 shown).

We successfully obtained 44 mitochondrial cytochrome *b* (*Cytb*) gene sequences, each with a final length of 1083 bp after alignment. Among these, 22 sequences were derived from *Sineuchiloglanis chishuiensis* sp. nov., comprising three distinct haplotypes, and the remaining 22 sequences from *S. baishuiensis* sp. nov. represented two unique haplotypes. These five haplotypes were subsequently analyzed together with 53 *Cytb* sequences downloaded from NCBI. Maximum likelihood phylogenetic analysis (Fig. 9) revealed that all specimens of *S. chishuiensis* sp. nov. and *S. baishuiensis* sp. nov. formed two separate, strongly supported monophyletic clades (bootstrap support = 100 for each clade).

Genetic divergence analysis based on the K2P model revealed distinct evolutionary relationships between the two newly described species and other members of *Sineuchiloglanis* (Table 5). Intraspecific genetic distance of *S. chishuiensis* sp. nov. and *S. baishuiensis* sp. nov. is 0%. The interspecific genetic distances of *S. chishuiensis* sp. nov. with its congeners ranged from 2.3% (vs *S. hupingshanensis*) to 14.5% (vs *S. chui*). Similarly, *S. baishuiensis* sp. nov. showed genetic distances varying from 1.8% (vs *S. hupingshanensis*) to 14.4% (vs *S. chui*) when compared with other *Sineuchiloglanis* species. Notably, the genetic divergence between the two new species ranged from 2.4% to 2.7%.

**Table 5.** Genetic distances of *Sineuchiloglanis* species from the Yangtze River, China.

	1	2	3	4	5	6	7	8	9
**1 *Sineuchiloglanis chishuiensis* sp. nov**.									
**2 *Sineuchiloglanis chishuiensis* sp. nov**.	0.002								
**3 *Sineuchiloglanis chishuiensis* sp. nov**.	0.001	0.003							
**4 *Sineuchiloglanis baishuiensis* sp. nov**.	0.024	0.026	0.025						
**5 *Sineuchiloglanis baishuiensis* sp. nov**.	0.025	0.027	0.026	0.001					
**6 *Sineuchiloglanis anteanalis***	0.027	0.029	0.028	0.028	0.029				
**7 *Sineuchiloglanis hupingshanensis***	0.023	0.025	0.024	0.018	0.019	0.027			
**8 *Sineuchiloglanis chui***	0.144	0.141	0.145	0.143	0.144	0.140	0.139		
**9 *Sineuchiloglanis robusta***	0.047	0.047	0.048	0.048	0.049	0.040	0.043	0.140	
**10 *Sineuchiloglanis sichuanensis***	0.047	0.047	0.048	0.048	0.049	0.040	0.043	0.140	0.000

##### Key to the species of *Sineuchiloglanis* in the Yangtze River in China

**Table:** 

1	Dorsal-fin rays i, 4^1/2^	**2**
–	Dorsal-fin rays i, 5^1/2^	**3**
2	Caudal-fin rays i, 6+7, i	***S. chui***
–	Caudal-fin rays i, 7+8, i	***S. hupingshanensis***
3	Gill opening extending to the base of fourth to fifth pectoral-fin elements	**4**
–	Gill opening extending to the base of second to third pectoral-fin elements	**5**
4	The ratio of maxillary barbel length to head length is greater than 6/7	***S. robusta***
–	The ratio of maxillary barbel length to head length is less than 6/7	***S. baishuiensis* sp. nov**.
5	The ratio of caudal depth to caudal length is less than 1/4	***S. anteanalis***
–	The ratio of caudal depth to caudal length is greater than 1/4	**6**
6	Nasal barbel tip not reaching anterior orbital	***S. chishuiensis* sp. nov**.
–	Nasal barbel tip extending beyond posterior orbital	***S. sichuanensis***

## Discussion

With the description of these two new species, the genus now comprises eleven recognized species: *S. myzostoma* Norman, 1923; *S. gracilicauda* (Wu & Chen, 1979); *S. anteanalis* Fang, Xu & Cui, 1984; *S. robusta* Ding, Fu & Ye, 1991; *S. sichuanensis* Ding, Fu & Ye, 1991; *S. nebulifera* Ng & Kottelat, 2000; *S. prolixdorsalis* Li, Zhou, Thomson, Zhang & Yang, 2007; *S. hupingshanensis* Kang, Chen & He, 2016; *S. chui* (Li, Dao & Zhou, 2020) with the two newly described taxa. The two new species described herein are readily distinguished from other *Sineuchiloglanis* species from the Yangtze River by well-defined characters (Tables 2, 4). The major characters of *Sineuchiloglanis chishuiensis* sp. nov. are dorsal-fin rays i, 5^1/2^; pectoral-fin rays i, 12–13; anal-fin rays i, 4^1/2^; caudal-fin rays i, 7+8, i; gill opening extending to the base of second and third pectoral-fin elements; tip of maxillary barbel not reaching or extending beyond the lower corner of the gill opening; tip of nasal barbel not reaching anterior orbital margin; pelvic-fin tip reaching or extending beyond anus; the ventral abdominal surface slightly convex. The major characters of *Sineuchiloglanis baishuiensis* sp. nov. are dorsal-fin rays i, 5^1/2^; anal-fin rays i, 4^1/2^; caudal-fin rays i, 7+8, i; gill opening extending to the base of fourth and fifth pectoral-fin elements; tip of maxillary barbel not reaching the lower corner of the gill opening; tip of nasal barbel not reaching anterior orbital margin; the ventral abdominal surface flattened. *S. chishuiensis* sp. nov. and *S. baishuiensis* sp. nov. are significantly distinguishable by the lower corner of the gill opening (opposite base of the second and third pectoral-fin elements in *S. chishuiensis* sp. nov. vs opposite base of the fourth and fifth pectoral-fin elements in *S. baishuiensis* sp. nov.), ventral abdominal surface (slightly convex lateral abdominal regions with a shallow medially invaginated groove along the ventral midline in *S. chishuiensis* sp. nov. vs flattened ventral abdominal surface in *S. baishuiensis* sp. nov.).

The two new species distinctly differ from *S. anteanalis* by the thicker caudal-peduncle (depth 27.1–40.3% of length in *S. chishuiensis* sp. nov. and 25.9–40.6% in *S. baishuiensis* sp. nov. vs 13.0–24.6% in *S. anteanalis*), more branched anal-fin rays i,4^1/2^(vs i, 3^1/2^), more caudal-fin rays i,7+8, i (vs i, 6+7, i), more vertebra number (13 + 25 = 38 (*n* = 1), 13 + 24 = 37 (*n* = 1), 12 + 24 = 36 (*n* = 2) in *S. chishuiensis* sp. nov. and 13 + 23 = 36 (*n* = 2), 13 + 24 = 37 (*n* = 2) in *S. baishuiensis* sp. nov. vs 12 + 21 = 33 (*n* = 1), 12 + 20 = 32 (*n* = 1), 11 + 21 = 32 (*n* = 2) in *S. anteanalis*), lower corner of gill opening (extending to opposite base of the fourth and fifth pectoral-fin elements in *S. baishuiensis* sp. nov. vs extending to opposite base of the second and third pectoral-fin elements in *S. anteanalis*).

These two species are distinct from *S. hupingshanensis* by the greater interorbital distance (24.1–28.8% of HL in *S. chishuiensis* sp. nov. and 22.8–29.6% in *S. baishuiensis* sp. nov. vs 20.4–23.8% in *S. hupingshanensis*), pectoral-fin depth (53.0–70.5% of pectoral-fin length in *S. chishuiensis* sp. nov. and 61.0–78.2% in *S. baishuiensis* sp. nov. vs 38.4–51.4% in *S. hupingshanensis*), pelvic-fin width (43.0–68.4% of pelvic-fin length in *S. chishuiensis* sp. nov. and 56.6–76.6% in *S. baishuiensis* sp. nov. vs 35.6–49.0% in *S. hupingshanensis*), head depth (38.9–53.1% of SL in *S. chishuiensis* sp. nov. and 42.0–48.4% in *S. baishuiensis* sp. nov. vs 37.2–41.1% in *S. hupingshanensis*), more branched dorsal-fin rays i, 5^1/2^ (vs i, 4^1/2^), lower corner of gill opening (extending to opposite base of the fourth and fifth pectoral-fin elements in *S. baishuiensis* sp. nov. vs extending to opposite base of the second and third pectoral-fin elements *S. hupingshanensis*), maxillary barbel tip (not reaching or extending beyond lower gill-opening corner in *S. chishuiensis* sp. nov. and not reaching the lower gill-opening corner in *S. baishuiensis* sp. nov. (vs extending beyond lower gill-opening corner in *S. hupingshanensis*), nasal barbel tip not reaching anterior orbital in both *S. chishuiensis* sp. nov. and *S. baishuiensis* sp. nov. (vs extending beyond anterior orbital in *S. hupingshanensis*), pelvic-fin tip (reaching or extending beyond anus in *S. chishuiensis* sp. nov. and not reaching, reaching, or extending beyond anus in S. baishuiensis sp. nov. vs not reaching anus in *S. hupingshanensis*).

The new species further differ from *S. chui* by the greater eye diameter (6.8–9.1% of HL in *S. chishuiensis* sp. nov. and 6.1–9.2% in *S. baishuiensis* sp. nov. vs 5.2–6.7% in *S. chui*), head length behind the eyes (35.6–40.3% of SL in *S. chishuiensis* sp. nov. and 35.4–39.4% in *S. baishuiensis* sp. nov. vs 40.4–45.5% in *S. chui*), pelvic-fin origin to anal-fin origin distance (24.4–32.4% of SL in *S. chishuiensis* sp. nov. vs 29.2–32.5% in *S. chui*), pectoral-fin width (61.0–78.2% of pectoral-fin length in *S. baishuiensis* sp. nov. vs 42.2–56.3% in *S. chui*), more branched dorsal-fin rays (i, 5^1/2^ in *S. chishuiensis* sp. nov. and *S. baishuiensis* sp. nov. vs i, 4^1/2^ in *S. chui*), and branched anal-fin rays (i, 4^1/2^ in *S. chishuiensis* sp. nov. and *S. baishuiensis* sp. nov. vs i, 3^1/2^ in *S. chui*), more vertebra number (13 + 25 = 38 (*n* = 1), 13 + 24 = 37 (*n* = 1), 12 + 24 = 36 (*n* = 2) in *S. chishuiensis* sp. nov. and 13 + 23 = 36 (*n* = 2), 13 + 24 = 37 (*n* = 2) in *S. baishuiensis* sp. nov. vs 12 + 21 = 33 (*n* = 1), 12 +20 = 32 (*n* = 1), 11 + 21 = 32 (*n* = 2) in *S. chui*), lower corner of gill opening (extending to opposite base of the fourth and fifth pectoral-fin elements in *S. baishuiensis* sp. nov. vs extending to opposite base of the second and third pectoral-fin elements in *S. chui*), nasal barbel tip not reaching anterior orbital in both *S. chishuiensis* sp. nov. and *S. baishuiensis* sp. nov. (vs extending beyond anterior orbital and not reaching posterior orbital in *S. chui*).

The new species further differ from *S. robusta* by the dorsal-fin insertion to adipose-fin origin distance (14.1–23.2% of SL in *S. chishuiensis* sp. nov. and 15.5–25.5% in *S. baishuiensis* sp. nov. vs 26.5–33.5% in *S. robusta*), maxillary barbel length (67.1–84.8% of HL in *S. chishuiensis* sp. nov. and 65.9–82.1% in *S. baishuiensis* sp. nov. vs 90.5–97.7% in *S. robusta*), head width (18.5–24.5% of HL in *S. chishuiensis* sp. nov. and 18.1–21.0% in *S. baishuiensis* sp. nov. vs 21.3–23.9% in *S. robusta*), less branched pectoral-fin rays (i, 12–13 in *S. chishuiensis* sp. nov. and i, 12–14 in *S. baishuiensis* sp. nov. vs i, 14–15 in *S. robusta*), a greater number of vertebra (13 + 25 = 38 (*n* = 1), 13 + 24 = 37 (*n* = 1), 12 + 24 = 36 (*n* = 2) in *S. chishuiensis* sp. nov. and 13 + 23 = 36 (*n* = 2), 13 + 24 = 37 (*n* = 2) in *S. baishuiensis* sp. nov. vs 12 + 21 = 33 (*n* = 1) in *S. robusta*), lower corner of gill opening (extending to opposite base of the second and third pectoral-fin elements in *S. chishuiensis* sp. nov. vs extending to opposite base of the fourth and fifth pectoral-fin elements in *S. robusta*).

The new species further differ from *S. sichuanensis* by the shorter dorsal-fin insertion to adipose-fin origin distance (14.1–23.2% of SL in *S. chishuiensis* sp. nov. and 15.5–25.5% in *S. baishuiensis* sp. nov. vs 25.2–28.4% in *S. sichuanensis*), shorter maxillary barbel length (67.1–84.8% of HL in *S. chishuiensis* sp. nov. and 65.9–82.1% in *S. baishuiensis* sp. nov. vs 91.1–96.2% in *S. sichuanensis*), greater caudal-peduncle length (21.5–29.2% of SL in *S. chishuiensis* sp. nov. and 20.6–26.72% in *S. baihsuijiangensis* sp. nov. vs 15.7–19.6% in *S. sichuanensis*), post-adipose length (8.2–12.7% of SL in *S. chishuiensis* sp. nov. and 8.1–11.2% in *S. baishuiensis* sp. nov. vs 4.6–7.6% in *S. sichuanensis*), a greater number of vertebra (13 + 25 = 38; *n* = 1), 13 + 24 = 37 (*n* = 1), 12 + 24 = 36 (*n* = 2) in *S. chishuiensis* sp. nov. and 13 + 23 = 36 (*n* = 2), 13 + 24 = 37 (*n* = 2) in *S. baishuiensis* sp. nov. vs 19 + 15 = 34 (*n* = 2) in *S. sichuanensis*), lower corner of gill opening (extending to opposite base of the fourth and fifth pectoral-fin elements in *S. baishuiensis* sp. nov. vs extending to opposite base of the second and third pectoral-fin elements in *S. sichuanensis*), maxillary barbel tip (not reaching or extending beyond lower gill-opening corner in *S. chishuiensis* sp. nov. and not reaching the lower gill-opening corner in *S. baishuiensis* sp. nov. vs reaching the lower gill-opening corner in *S. sichuanensis*), nasal barbel tip not reaching anterior orbital in both *S. chishuiensis* sp. nov. and *S. baishuiensis* sp. nov. (vs extending beyond anterior orbital in *S. sichuanensis*).

Molecular phylogenetic evidence corroborated the validity of *S. chishuiensis* sp. nov. and *S. baishuiensis* sp. nov. by the highly supported monophyly and sufficient genetic divergence from their congeners. Meanwhile, the genetic distance between *S. baishuiensis* sp. nov. and *S. hupingshanensis* was slightly below the 2% threshold, suggesting that their differentiation was at an early stage. Meanwhile, it should be noted that *S. chishuiensis* sp. nov., *S. baishuiensis* and *S. robusta* are more analogous in morphology (dorsal-fin ray i, 5^1/2^, anal-fin ray i, 4^1/2^, caudal-fin ray i,7+8, i, nasal barbel tip not reaching anterior orbital, for instance), while the topology of the phylogenetic tree demonstrated that these species clustered neither by the river system pattern nor by the morphological analogousness.

According to Chu (1981), who restored *Pareuchiloglanis* with *P. poilanei* Pellegrin, 1936 as type species, *Pareuchiloglanis* is different from Euchiloglanis (Chimarrichthys) in that the sides of the premaxillary tooth band do not extend backwards. Thereafter, all species with homodont dentition in narrow bands with sides not extending backwards were classified as *Pareuchiloglanis*. Subsequent molecular phylogenetic studies, however, demonstrated that *Pareuchiloglanis* was not monophyletic, but rather had a number of morphologically distinct species groups that typically exhibit a geographical pattern (Peng et al. 2004, 2006; Guo et al. 2004, 2005, 2007; Yu and He 2012; Ma et al. 2015; Dai et al. 2021). Subsequent research has further raised these species groups into several genera, including *Creteuchiloglanis*, *Barbeuchiloglanis*, *Sineuchiloglanis*, and *Tremeuchiloglanis* (Zhou et al. 2011; Li et al. 2022). Along with *Barbeuchiloglanis* and *Tremeuchiloglanis*, the genus *Sineuchiloglanis* is one of the three new genera described in revision of *Pareuchiloglanis* (Li et al. 2022). However, phylogenetic analysis based on mitochondrial *Cytb* genes does not yet support the monophyly of *Sineuchiloglanis*, *Barbeuchiloglanis*, and *Tremeuchiloglanis*, displaying species from multiple genera nesting with one another (Fig. 9). In the most extreme cases, species of different genera share the same haplotype (e.g., OM428184 of *T. rhabdurus*, collected from Mengdong, Malipo County, Yunnan Province, China and OM428188 of *S. chui*, collected from Judian, Yulong County, Yunnan Province). Similar phenomena also occur between different species of *Sineuchiloglanis* (e.g., OM428192 of *S. robusta*, collected from Ebian, Sichuan Province, and OM428193 of *S. sichuanensis*, collected from Yajiang, Sichuan Province). Possible reasons for this chaotic phylogeny include incomplete lineage sorting, failure of the mitochondrial gene to reflect phylogenetic relationships, extensive interspecific hybridization and introgression, and ancestral polymorphism (Funk and Omland 2003). In conclusion, additional evidence is required to support the validity of *Barbeuchiloglanis*, *Tremeuchiloglanis*, and *Sineuchiloglanis*. Future revisions to the taxonomy of glyptosternoid fishes will require a thorough collection of voucher specimens, the acquisition of molecular phylogenetic data across Glyptosterninae, and the selection of critical diagnostic characteristics for morphological comparisons.

## Comparative material

*Sineuchiloglanis hupingshanensis*: Institute of Hydrobiology, Chinese Academy of Sciences (IHB) 070819426, 070819429, 070819430, 071130392, 112.08–130.21 mm SL, China: Hunan Province: Shimen County: Hupingshan Town: Qingshi Village, 2007; 20080622224, 78.17 mm SL, China: Hunan Province: Shimen County: Hupingshan Town: Qingshi Village, 2008.

*S. chui*: Institute of Hydrobiology, Chinese Academy of Sciences (IHB) 0403168, 0403169, 0403170, 0403194, 0403195, 0403199, 109.01–150.66 mm SL, China: Sichuan Province: Garzê Tibetan Autonomous Prefecture: Jiulong County, 2004.

*S. anteanalis*: Institute of Hydrobiology, Chinese Academy of Sciences (IHB) 66-0501, 66-0496, 66-0502, 66-0503, 66-0504, 66-0506, 66-0507, 66-0508, 66-0512, 82.17–122.73 mm SL, China: Gansu Province: Gannan Tibetan Autonomous Prefecture: Zhouqu County, 1959; 0407054, 0407129, 0407158, 98.23–125.59 mm SL, China: Sichuan Province: Liangshan Yi Autonomous: Mianning County, 2004; 0509402, 0509403, 0509404, 0509405, 0509406, 0509407, 0509408, 0509409, 0509415, 0509416, 0509418, 0509420, 0509434, 82.17–144.78 mm SL, China: Sichuan Province: Liangshan Yi Autonomous: Mianning County, 2005.

*S. sichuanensis*: data from Li et al. (2020).

*S. robusta*: data from Li et al. (2020); Institute of Hydrobiology, Chinese Academy of Sciences (IHB) 0403190, 118.56 mm SL, China: Sichuan Province: Garzê Tibetan Autonomous Prefecture: Jiulong County, 2004.

## Supplementary Material

XML Treatment for Sineuchiloglanis chishuiensis

XML Treatment for Sineuchiloglanis baishuiensis

## Additional information

### Use of AI

No use of AI was reported.

### Author ORCIDs

Hai-Min Lv https://orcid.org/0009-0009-7713-6022

De-Kui He https://orcid.org/0000-0002-4001-826X

Fei Liu https://orcid.org/0000-0003-4404-0971

### Data availability

All of the data that support the findings of this study are available in the main text.
